# Treatment of lean and diet-induced obesity (DIO) mice with a novel stable obestatin analogue alters plasma metabolite levels as detected by untargeted LC–MS metabolomics

**DOI:** 10.1007/s11306-016-1063-0

**Published:** 2016-07-05

**Authors:** Elaine Cowan, Praveen Kumar, Kerry J. Burch, David J. Grieve, Brian D. Green, Stewart F. Graham

**Affiliations:** Institute for Global Food Security, Queen’s University of Belfast, Belfast, BT9 5BN Northern Ireland, UK; Wellcome-Wolfson Institute for Experimental Medicine, Queen’s University of Belfast, Belfast, BT9 7AE Northern Ireland, UK; Beaumont Research Institute, 3811 W. 13 Mile Road, Royal Oak, MI 48073 USA

**Keywords:** Obestatin, Nutrition, Obesity, Diabetes, UPLC–MS, Metabolomics

## Abstract

**Introduction:**

Obestatin is a controversial gastrointestinal peptide purported to have metabolic actions.

**Objectives:**

This study investigated whether treatment with a stable obestatin analogue (PEG-OB(Cys^10^, Cys^13^)) changed plasma metabolite levels firstly in lean and subsequently in diet-induced obesity (DIO) C57BL6/J mice.

**Methods:**

Untargeted LC-HRMS metabolomics experiments were carried out in ESI + mode with plasma extracts from both groups of animals. Data were normalised, multivariate and univariate statistical analysis performed and metabolites of interest putatively identified.

**Results:**

In lean mice, 39 metabolites were significantly changed by obestatin treatment and the majority of these were increased, including various C16 and C18 moieties of phosphatidylcholine, phosphatidylethanolamine, phosphatidylserine and monoacylglycerol, along with vitamin A, vitamin D3, tyrosine, acetylcarnitine and 2α-(hydroxymethyl)-5α-androstane-3β,17β-diol. Decreased concentrations of glycolithocholic acid, 3-dehydroteasterone and various phospholipids were observed. In DIO mice, 25 metabolites were significantly affected and strikingly, the magnitudes of changes here were generally much greater in DIO mice than in lean mice, and in contrast, the majority of metabolite changes were decreases. Four metabolites affected in both groups included glycolithocholic acid, and three different long-chain (C18) phospholipid molecules (phosphatidylethanolamine, platelet activating factor (PAF), and monoacylglycerol). Metabolites exclusively affected in DIO mice included various phosphatidylcholines, lysophosphatidylcholines and fatty acyls, as well as creatine and oxidised glutathione.

**Conclusion:**

This investigation demonstrates that obestatin treatment affects phospholipid turnover and influences lipid homeostasis, whilst providing convincing evidence that obestatin may be acting to ameliorate diet-induced impairments in lipid metabolism, and it may influence steroid, bile acid, PAF and glutathione metabolism.

**Electronic supplementary material:**

The online version of this article (doi:10.1007/s11306-016-1063-0) contains supplementary material, which is available to authorized users.

## Introduction

Obestatin is a metabolic hormone recently discovered in rat stomach and initially described to inhibit food intake, decrease intestinal motility and curb body weight gain via the GPR39 G-protein coupled receptor. Its name is derived from two Latin words “obedere” meaning to devour and “statin” meaning to suppress. This 23 amino acid peptide hormone was originally thought to be a direct opponent of ghrelin, a peptide well characterised as an orexigenic hormone which negatively impacts glucose homeostasis; indeed, both obestatin and ghrelin are produced from post-translational modification of the same preproghrelin peptide encoded from the ghrelin gene (Dezaki 2013; Zhang et al. 2005). Primarily produced in the stomach, obestatin is also expressed in pancreatic and adipose tissue, skeletal muscle, liver, lung, thyroid, mammary glands and testes, suggesting it may be multifaceted in its functions and play autocrine/paracrine roles (Gesmundo et al. 2013; Seim et al. 2011). It has been found to act both centrally and peripherally with documented central effects most often relating to influence on food intake. The peptide has also been shown to inhibit thirst, regulate sleep patterns, and to influence anxiety levels and memory function actions which are thought to occur via stimulation of the vagal afferent pathway (Ataka et al. 2008; Carlini et al. 2007; Samson et al. 2007; Szentirmai and Krueger 2006). Peripheral effects have frequently been reported in the pancreas and adipose tissue, with additional actions also documented in the cardiovascular system and skeletal muscle (Agnew et al. 2012; Trovato et al. 2014).

Among the many gut hormones, obestatin is particularly controversial due to a lack of experimental reproducibility in its reported actions, which may in part be attributed to its short biological half-life in vivo (Vergote et al. 2008). Thus far, effects of obestatin on feeding behaviour, body weight and gastrointestinal activity have both been confirmed and refuted in numerous studies. Furthermore, any interaction with the GPR39 receptor has been refuted to the extent that obestatin is now widely considered to be an orphan ligand. Nevertheless, there is growing interest in obestatin’s apparent actions on glucose and lipid metabolism (Gargantini et al. 2013) as well as its ability to promote proliferation and to inhibit apoptosis in pancreatic β-cells and adipocytes (Granata et al. 2008; Tang et al. 2014). For example, there is evidence that obestatin modulates the expression of adipogenic and glucoregulatory genes and that it modulates insulin secretion, adipocyte GLUT-4 translocation, free fatty acid uptake, and glucose uptake and may inhibit lipolysis in white adipose tissue (Favaro et al. 2012; Granata et al. 2008, 2012; Tang et al. 2014). Although some findings are contradictory (Pruszynska-Oszmalek et al. 2013; Ren et al. 2013), it is becoming clear that plasma obestatin levels are disturbed following the development of obesity and diabetes (Seim et al. 2011; Trovato et al. 2014). Indeed, a growing body of evidence suggests that obestatin could play a positive role in obesity and obesity-induced diabetes.

Metabolomics is a systems biology technique used to investigate changes in the abundance of low molecular weight metabolites in tissues and biofluids and has been used to interrogate the mechanisms underlying many diseases and therapies (Bain et al. 2009). Indeed, a number of targeted and untargeted metabolomics studies have been conducted to examine the effect of obesity/diabetes on the metabolome, as well as the influence of clinically approved anti-diabetic therapies (Bao et al. 2009; Duggan et al. 2011; Park et al. 2015). This has provided new insights into the aetiology of obesity and diabetes and has pinpointed specific disturbances in amino acid, lipid, carbohydrate and nucleotide metabolism (Park et al. 2015).

Here, we examined the effects of obestatin treatment on the plasma metabolome. Our first aim was to assess the controversial status of obestatin as a hormone by establishing if 6 weeks of daily treatment affects metabolite levels (thereby indicating if there are modulatory effects on metabolism) in normal mice. Once this was verified the second aim was to then determine whether the observed obestatin-mediated effects on metabolism were substantially different in mice subjected to diet-induced obesity (DIO). Since the reported actions of obestatin are extremely diverse, potentially with more actions remaining undiscovered, this investigation focused on peripheral blood metabolites and employed an untargeted UPLC-MS metabolomics methodology. Importantly, since obestatin is physiologically degraded the treatment involved the use of a novel stable obestatin analogue resistant to degradation.

## Materials and methods

### Animals and sampling

C57BL6/J male mice were bred in-house, maintained under constant climatic conditions at 21 °C with 12:12 h light–dark cycles, housed either individually or in pairs and given free access to food and water. All experiments were performed in accordance with the Guidance on the Operation of the Animals (Scientific Procedures) Act, 1986 and approved by the Queen’s University Belfast Animal Welfare and Ethical Review Body. Lean mice (n = 12) received a standard laboratory diet, composed of 16.4 % protein, 4.0 % fat and 48.5 % carbohydrate (3.0 kcal/g; Teklad global rodent diet, Envigo, UK) for 23 weeks from birth. For the final 6 weeks animals were injected with either saline (n = 6) or a modified obestatin peptide N-PEGylated(Cys^10^, Cys^13^)obestatin (n = 6; PEG-OB(Cys^10^, Cys^13^)) (GL Biochem, China) at 50 nmol/kg/day. Injections were administered subcutaneously in the back of the neck at a volume of 10 ml/kg and the final weight range of the animals in each group was 25–33 g (saline) and 26–33 g (PEG-OB(Cys^10^, Cys^13^)).

In a separate experiment the effects of PEG-OB(Cys^10^, Cys^13^) were investigated in DIO mice (n = 11) which received a standard laboratory diet until 4 weeks of age, thereafter they received a high fat diet (20.5 % protein, 36.0 % fat and 35.7 % carbohydrate (5.49 kcal/g; Mouse Diet, 60 % Fat Calories, BioServ, USA) for 19 weeks. For the final 6 weeks, mice were separated into two groups and injected with either saline (n = 5) or PEG-OB(Cys^10^, Cys^13^) (n = 6) at 50 nmol/kg/day as for lean mice. The final weight range of the DIO animals in each group was 39–55 g (saline) and 42–56 g (PEG-OB(Cys^10^, Cys^13^)). At the end of the 23 week period, mice were fasted for 5 h and sacrificed by CO_2_ asphyxiation 3 h after the onset of the light cycle and exactly 1 h after injection on the final day of treatment. Blood obtained by cardiac puncture was collected into pre-chilled heparinised tubes, centrifuged at 3000×*g* for 10 min (4 °C) and the resulting plasma transferred to W/PTFE lined vials (Supelco, USA) prior to storage at −20 °C for metabolomic analysis.

### Sample preparation

Frozen plasma aliquots were thawed on ice and 100 µl was added to 300 µl of ice cold methanol (100 %) in a 2 ml sterile Eppendorf tube. Samples were mixed for 30 s and the protein removed by centrifugation at 13,000×*g* for 15 min (4 °C). Supernatants were evaporated to dryness, reconstituted in 100 µl of ultra-pure water (Millipore) and filtered by centrifugation using a 0.22 µm Costar spin-X centrifuge tube filter (8000×*g* at 4 °C for 5 min; Corning Incorporated, Corning, NY 14831, USA).

### UPLC-MS analysis

All solvents were purchased from Fisher Scientific (Pittsburg, USA) and were LC-MS grade or equivalent. Chromatography was performed on a Dionex Ultimate 3000 UHPLC system (Dionex, Softron GmbH, Germany) coupled to an LTQ Orbitrap Elite mass spectrometer (Thermo Fisher Scientific, Bremen, Germany). 5 µl of extracted plasma was injected (n = 3 injections for each sample) onto an Acquity UPLC CSH C18 column (2.1 × 100 mm, 1.7 µm, Waters, Wexford, Ireland) operating at 50 °C and applying a binary mobile phase system. The sample manager temperature was maintained at 4 °C and the order in which the samples were injected was randomised throughout the experiment. The gradient elution buffers were A (water with 0.1 % formic acid (vol/vol)) and B (methanol with 0.1 % formic acid (vol/vol)). Solvent B was varied as follows: 0 min 1 %, 2.5 min 1 %, 16 min 99 %, 18 min 99 %, 18.1 min 1 % and 20 min 1 % with a flow rate of 0.4 ml min^−1^. Positive ionisation mode was employed with these conditions; source heater temperature at 400 °C, sheath gas at 60 (AU), aux gas at 45 (AU) and sweep gas at 1 (AU), capillary temp was maintained at 325 °C and source voltage at 3.5 kV. Mass spectra data were acquired in profile mode over the 50–1200 *m/z* range with a mass resolution of 60,000 at mass 400 (FWHM) and a scan time of 0.5 s. In further experiments, the samples were subjected to mass fragmentation analysis (FT HCD (10, 30 and 70 NCE), MS^2^) with an isolation width of 1 Da and 60,000 FWHM at 400 m/z.

Initially the mass spectrometer was calibrated using LTQ Velos ESI Positive Ion Calibration Solution (Thermo Scientific) and a mixture of metabolites (100 µg/ml uridine, nicotinic acid, tryptophan, hippuric acid and phenylalanine; ACROS organics) in water. Prior to sample analysis 10 pooled conditioning samples were injected. To determine chromatographic reproducibility of retention times and peak intensities, pooled samples were injected after every 10 sample injections throughout the experiment (Graham et al. 2013; Want et al. 2010). These pooled samples comprised of plasma from this study and a separate similar sized pharmacological study (C57BL6/J male mice).

### Data analysis

UPLC-MS acquired data were analysed using Progenesis QI software (Waters Corporation, Milford, MA) for peak alignment, peak picking and data normalisation. A peak threshold filter of 2.5 AU was applied and peak picking thresholds were set between 0.5 and 20 min. Data were normalised to all compounds by correcting for multiple features to determine a global scaling factor. An output table was subsequently generated to include paired *m/z* retention times and raw and normalised peak intensities for pools and individual samples. These metabolic features were exported to Simca P v.14 (Umetrics, Umea, Sweden) for multivariate analysis by principal component analysis (PCA) and data quality was assessed via visualisation of clustering of pools and sample replicates. PCA observations were indicative of good platform stability (Supplementary Figure 1). UPLC-MS acquired data for all samples (lean and DIO) were subsequently reanalysed using Progenesis QI as previously described.

On the basis of normalised peak intensities, metabolic features were reduced by exclusion of those observations showing no change in magnitude (fold change = 1) and no significant statistical difference (*P* > 0.05, students *t* test). Metabolic features were reduced from 3819 to 736 and 3819 to 615 when PEG-OB(Cys^10^, Cys^13^) and saline treated mice were compared within lean and DIO groups, respectively. This was carried out to avoid over-fitting and to improve the model’s predictive ability. Using the average value for each biological replicate the filtered metabolic features were exported to Simca 14 Simca P v.14 (Umetrics, Umea, Sweden) for multivariate analysis. Data were mean centred, pareto scaled and grouped into PEG-OB(Cys^10^, Cys^13^) and saline treated groups for analysis using PCA and orthogonal projection to latent structures via partial least squares discriminant analysis (OPLS-DA). The S-plots were used to highlight the ions with the greatest influence on the separation between PEG-OB(Cys^10^, Cys^13^) and saline treated animals.

Overall model performance was assessed by R2 indicative of variation described by all components in the model and by Q2, measuring the models ability to predict class membership. The latter is made possible by the sevenfold cross validation default method of the SIMCA software whereby 1/7th of the data from the model is omitted and then predicted for class membership. Further to this, in each case a predictive model was built with the original data from 2/3 of the animals (training set) and used to blindly predict the remaining 1/3 (test set). Overall, the test set observations were assigned to the correct group in 87.5 % of cases. Permutation testing (n = 999) was also carried out to determine if the models were accurate i.e. the model fits the training set well and predicts Y accurately for new observations (Graham et al. 2016).

Discriminatory metabolites as highlighted in the S plot were investigated by using exact mass when searching HMDB (Wishart et al. 2009, 2013; Wishart et al. 2007) (http://www.hmdb.ca/) and the Metlin library (https://metlin.scripps.edu/) online databases. Putative identifications were further examined to increase confidence in identification by mass fragmentation analyses whereby ms/ms spectra were used to search spectral libraries via additional online databases *m/z* cloud (https://www.mzcloud.org/; http://www.highchem.com/) and MetFrag (http://msbi.ipb-halle.de/MetFrag/).

## Results

### Multivariate analysis

**Fig. 1:**
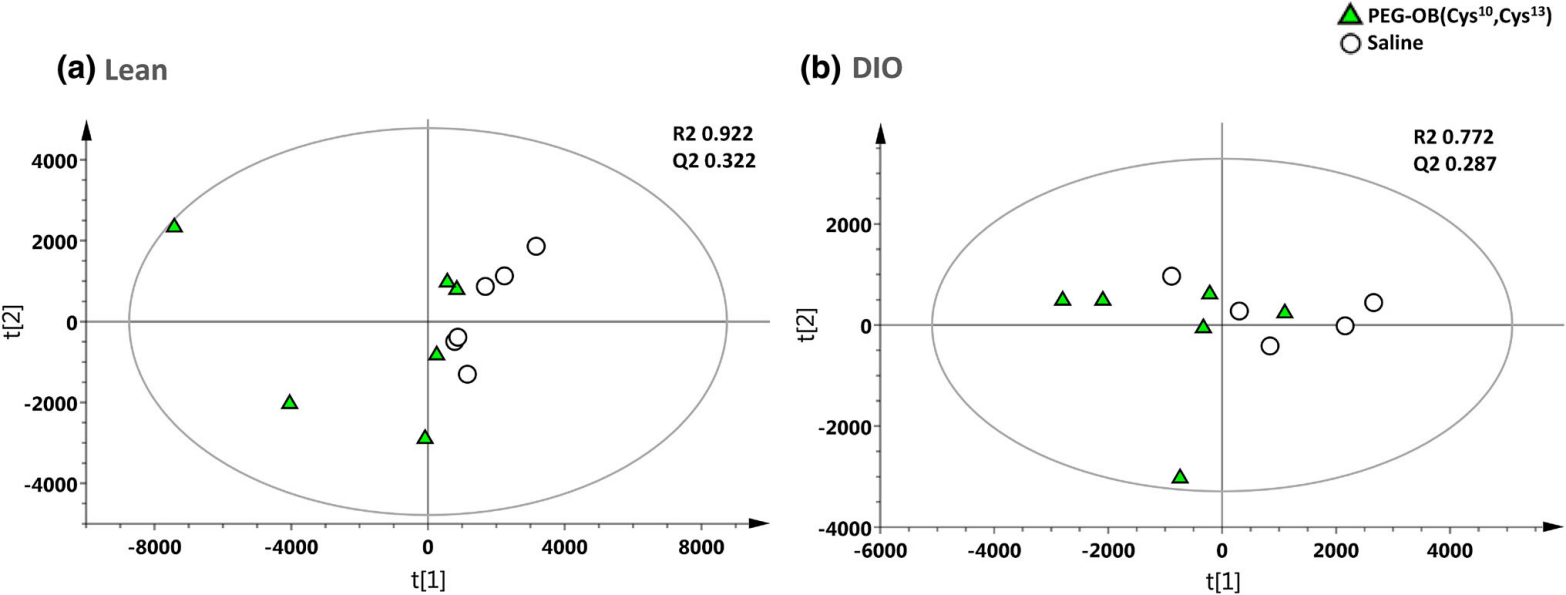
PCA scores plot displaying the separation for lean mice (**a**) and DIO mice (**b**) treated daily with PEG-OB(Cys^10^,Cys^13^) (*triangles*) or with saline (*circles*) for 6 weeks. Explained variances (R2) were 0.922 and 0.772 and predictive abilities (Q2) were 0.322 and 0.287 for lean and DIO mice respectively

**Fig. 2:**
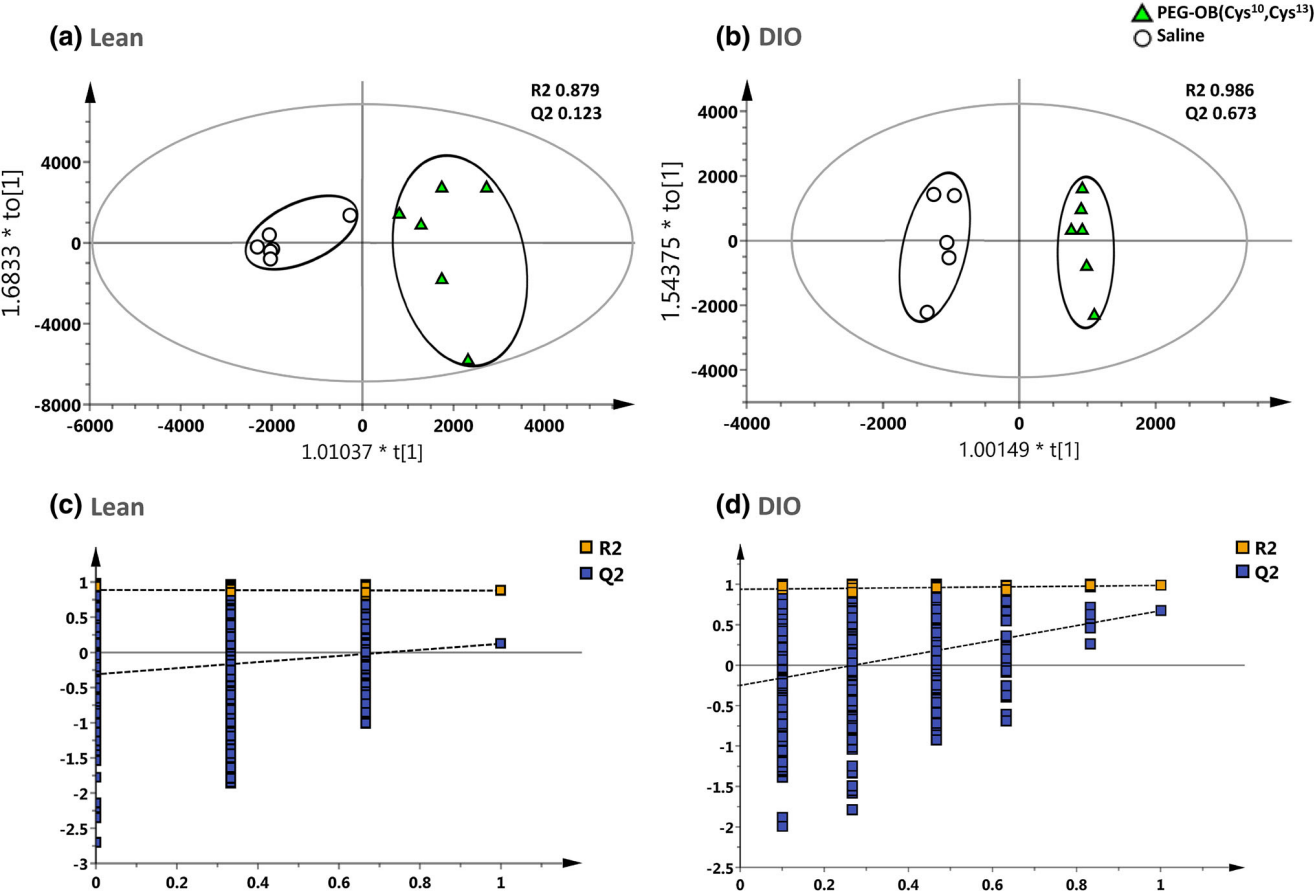
OPLS-DA scores plot displaying the separation for lean mice (**a**) and DIO mice (**b**) treated daily with PEG-OB(Cys^10^, Cys^13^) (*triangles*) or with saline (*circles*) for 6 weeks. Explained variances (R2) were 0.879 and 0.986 and predictive abilities (Q2) were 0.123 and 0.673 for lean and DIO mice respectively. Corresponding validation plots for lean mice (**c**) and DIO mice (**d**) displaying 999 permutation tests for model built from PEG-OB(Cys^10^, Cys^13^) treated animals

Figure 1 displays the PCA analysis of the raw mass spectral data acquired from PEG-OB(Cys^10^, Cys^13^) and saline treated plasma extracts using selected ion features (*P* < 0.05 and fold change ≠ 1). Figure 1a displays the PCA scores plot for lean mice and Fig. 1b displays the PCA scores plot for DIO mice. Both scores plots represent each group treated daily with PEG-OB(Cys^10^,Cys^13^) (triangles) or with saline (circles) for a period of 6 weeks. The PCA scores plots show subtle to moderate separation between respective classes. Subsequently OPLS-DA was used to build predictive models capable of discriminating between PEG-OB(Cys^10^, Cys^13^) (triangles) and saline (circles) treated animals (n = 3 replicates per sample) with increased power. Figure 2a and 2b display the OPLS-DA scores plots for PEG-OB(Cys^10^, Cys^13^) and saline treated animals in both lean and DIO groups, respectively. The scores plot for each model demonstrates clear separation between the two groups. R2 and Q2 values reflecting the goodness of fit and the predictive abilities of the models were 0.879 and 0.123 (1 orthogonal and 2 latent components) for the comparison in lean animals and 0.986 and 0.673 (1 orthogonal and 3 latent components) for the comparison in DIO animals, respectively. These values signify minimal model variance for both groups, and low to moderate predictive ability for lean and DIO groups respectively. These observations are further highlighted in Fig. 2c and 2d where the permutation plots (n = 999) of the OPLS-DA models are displayed for both sets of data respectively.

**Fig. 3:**
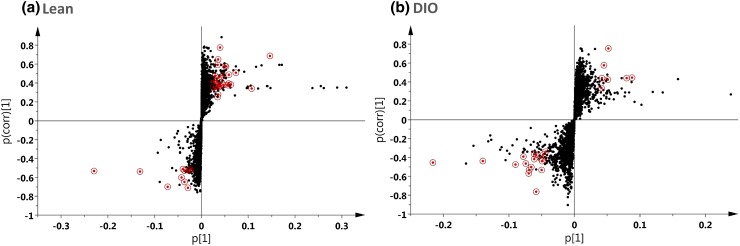
S-Plot displaying ions of interest (*circled*) for lean (**a**) and DIO (**b**) mice. Ions of interest were selected from 736 and 615 of the original 3819 metabolic features for lean and DIO mice respectively. These 736 and 615 ions demonstrated differences in magnitude (fold change ≠ 1) and significance (*P* < 0.05) between PEG-OB(Cys^10^, Cys^13^) and saline treated animals

S-plots as demonstrated by Fig. 3a and b were generated to identify the metabolites contributing to the discrimination between PEG-OB(Cys^10^, Cys^13^) and saline treated animals for both lean and DIO groups respectively. Metabolites with both higher and lower values for pcorr and p(corr) (1) were those increased and decreased with PEG-OB(Cys^10^, Cys^13^) treatment respectively. These were the most significant for differentiating between PEG-OB(Cys^10^, Cys^13^) and saline treated animals and are highlighted by circling on the s-plots. Note, that some ions with high pcorr and p(corr) (1) values are not highlighted on the s-plots. These ions had large changes in magnitude when comparing PEG-OB(Cys^10^, Cys^13^) and saline treated groups but were not significantly different due to the presence of clear outliers.

Multivariate analysis of technical replicates reported as Supplementary Figures 2 and 3 produced PCA and OPLS-DA scores plots for both data sets which showed good clustering of samples. Furthermore these data mirrored the scores plots acquired using the averaged biological replicates. The models produced using the larger technical replicate data set had greater R2, Q2 and better permutation results using the same ions of interest. These observations confirm the validity of the original model using biological replicates for ion selection and furthermore verify that using a larger sample size would produce better predictive models.

### Metabolite profile changes with PEG-OB(Cys^10^, Cys^13^) obestatin treatment

Of the original n = 3819 metabolic features, 39 and 25 were tentatively assigned from comparison of PEG-OB(Cys^10^, Cys^13^) and saline treated animals in lean and DIO groups, respectively (Tables 1, 2). Data presented for each metabolic feature in Tables 1 and 2 include putative identifications, mass to charge ratio and retention time, empirical formula, adduct, deviation from exact mass, significance value, false discovery rate, percentage increase, and area under the curve (ROC analysis) values. Among the documented putatively identified metabolites, 29 were significantly increased (Table 1a) and 10 significantly decreased (Table 1b) with PEG-OB(Cys^10^, Cys^13^) treatment in lean mice, whilst 7 were significantly increased (Table 2a) and 18 were significantly decreased (Table 2b) in DIO mice. All of these metabolites also remained significantly altered after *P* value adjustment by false discovery rate (FDR), and furthermore, area under the curve values for all metabolites by ROC analysis were >0.6.

**Table 1:** (a) Circulating metabolites increased in lean mice treated daily with PEG-OB(Cys^10^, Cys^13^) for 6 weeks, (b) Circulating metabolites decreased in lean mice treated daily with PEG-OB(Cys^10^, Cys^13^) for 6 weeks

Putative ID	RT(min)_m/z	Formula	Adduct	Δ ppm	*P* value	FDR	% change	AUC (ROC)	Identifier^d^
(*a*)									
Phospholipids									
PC(16:0/20:4(5Z,8Z,10E,14Z)(12OH[S]))^b^	16.75_820.5457	C44H80NO9P	M + Na	0	5.96E−03	1.55E−02	135.1	0.72	MID82375
PC(16:0/20:4(6E,8Z,11Z,14Z)(5OH[S]))^b^	16.52_798.5638	C44H80NO9P	M + H	0	1.96E−02	3.29E−02	187.2	0.65	MID82376
PC(O-16:1(9Z)/2:0)[U]^b^	14.91_544.3375	C26H52NO7P	M + Na	0	4.74E−03	1.32E−02	8.5	0.77	MID40010
PC(16:1(9Z)/2:0)^b^	13.12_536.3323	C26H50NO8P	M + H	4	2.46E−02	3.43E−02	123.0	0.63	MID39434
PC(17:0/18:3(9Z,12Z,15Z))^b^	16.94_802.5934	C43H80NO8P	M + CH3OH +H	2	2.78E−02	3.74E−02	83.5	0.71	MID75787
1-Palmitoyl-2-epoxystearoylphosphatidylcholine^b^	16.72_798.5625	C42H82NO9P	M + Na	0	1.69E−03	6.61E−03	78.9	0.77	None
LysoPC(20:4(5Z,8Z,11Z,14Z))^b^	14.77_544.3381	C28H50NO7P	M + H	3	2.19E−02	3.29E−02	10.3	0.71	HMDB10395
PE(20:2(11Z,14Z)/22:5(4Z,7Z,10Z,13Z,16Z))^b^	16.99_850.5935	C47H80NO8P	M + CH3OH +H	2	8.60E−03	1.77E−02	139.8	0.72	HMDB09307
PE(18:2((9Z,11Z)/18:2(9Z,11Z))[U]^b^	16.73_772.5472	C41H74NO8P	M + CH3OH +H	1	4.79E−02	4.79E−02	297.7	0.66	MID40429
PE(18:0/22:5(4Z,7Z,10Z,13Z,16Z))^b^	17.06_826.5934	C45H80NO8P	M + CH3OH +H	2	7.07E−03	1.62E−02	117.2	0.76	HMDB09010
PE(18:0/20:4(5Z,8Z,11Z,14Z))^b^	16.73_800.5777	C43H78NO8P	M + CH3OH +H	2	2.14E−02	3.29E−02	58.4	0.76	HMDB09003
PS(P-18:0/18:1(9Z))^b^	16.68_774.5631	C42H80NO9P	M + H	1	1.03E−02	2.01E−02	62.1	0.73	MID78831
PS(18:0/22:4(7Z,10Z,13Z,16Z))^b^	16.70_822.5625	C46H82NO10P	M + H-H2O	2	7.46E−03	1.62E−02	74.6	0.73	MID78547
Monoacylglycerols									
MG(18:1(9Z)/0:0/0:0)^b^	15.80_339.2911	C21H40O4	M + H-H2O	3	3.83E−03	1.24E−02	42.4	0.76	HMDB11567
MG(0:0/16:0/0:0)^b^	15.68_313.2750	C19H38O4	M + H-H2O	2	7.00E−03	1.62E−02	27.5	0.78	HMDB11533
Vitamins									
Vitamin D3^b^	16.36_367.3364	C27H44O	M + H-H2O	0	3.93E−02	4.72E−02	142.3	0.64	HMDB00876
25-Hydroxyvitamin-D3^b^	16.46_401.3413	C27H44O2	M + H	0	4.67E−02	4.79E−02	271.1	0.58	HMDB05997
Vitamin D3 derivative^c^	16.04_521.4201	C31H52O4	M + CH3OH +H	0	6.74E−04	3.76E−03	54.0	0.84	Several
9(cis)-retinal (Vitamin A)^a^	14.33_285.2207	C20H28O	M + H	2	4.69E−02	4.79E−02	112.3	0.68	HMDB06218
Fatty acids									
α-Linolenic acid^b^	14.05_279.2312	C18H30O2	M + H	2	4.41E−02	4.79E−02	22.2	0.68	HMDB01388
20-Hydroxyeicosatetraenoic acid^b^	14.33_343.2243	C20H32O3	M + Na	0	3.21E−02	4.18E−02	133.7	0.66	HMDB05998
5,8,11-Eicosatriynoic acid^b^	13.89_301.2156	C20H28O2	M + H	1	3.76E−02	4.72E−02	144.5	0.67	MID35294
5Z,8Z,11Z,14Z,17Z-Eicosapentaenoic acid^b^	14.33_303.2314	C20H30O2	M + H	1	4.24E−02	4.79E−02	108.8	0.67	HMDB01999
Amino acid, carnitine, steroid and unknowns									
Tyrosine^b^	1.30_182.0809	C9H11NO3	M + H	1	1.17E−08	4.55E−07	22.8	0.98	HMDB00158
Acetylcarnitine^a^	0.95_204.1221	C9H17NO4	M + H	4	4.49E−05	6.72E−04	15.6	0.85	HMDB00201
2α-(Hydroxymethyl)-5α-androstane-3β,17β-diol^b^	15.43_355.2843	C20H34O3	M + CH3OH +H	0	4.49E−02	4.79E−02	34.9	0.75	MID70469
None	10.19_394.2219	N/A	N/A	N/A	2.31E−02	3.34E−02	53.8	0.73	N/A
None	15.68_331.2864	N/A	N/A	N/A	1.35E−02	2.50E−02	19.3	0.73	N/A
None	15.80_357.3018	N/A	N/A	N/A	3.66E−03	1.24E−02	40.3	0.71	N/A
(*b*)									
Phospholipids									
LysoPC(22:0)^b^	16.70_580.4318	C30H62NO7P	M + H	3	1.07E−03	5.24E−03	−22.3	0.79	HMDB10398
PC(18:1(9Z)/20:4(5Z,8Z,11Z,14Z))^b^	17.29_830.5675	C46H82NO8P	M + Na	0	3.99E−02	4.72E−02	−11.6	0.71	HMDB08114
PAF C-18^b^	16.32_552.401	C28H58NO7P	M + H	2	2.20E−02	3.29E−02	−18.9	0.71	MID43410
LysoPC or PC^c^	17.03_608.4627	C32H66NO7P	M + H	3	4.51E−03	1.32E−02	−15.7	0.81	Several
LysoPE(0:0/20:3(5Z,8Z,11Z))^b^	14.93_526.2902	C25H46NO7P	M + Na	0	1.38E−04	1.34E−03	−34.4	0.77	HMDB11485
PE(18:2(9Z,12Z)/0:0)^b^	14.54_500.2756	C23H44NO7P	M + Na	1	5.17E−05	6.72E−04	−42.1	0.89	MID7767
PE(18:1(9Z)/0:0)^b^	14.80_502.2914	C23H46NO7P	M + Na	2	2.00E−02	3.29E−02	−22.4	0.65	MID40778
Sterol lipids and unknown									
Glycolithocholic acid^b^	14.81_478.2915	C26H43NO4	M + 2Na-H	2	3.89E−04	3.04E−03	−25.6	0.83	HMDB00698
3-Dehydroteasterone^b^	16.86_447.3469	C28H46O4	M + H	0	6.58E−04	3.76E−03	−17.9	0.82	HMDB41527
None	11.82_616.1743	N/A	N/A	N/A	1.48E−03	6.42E−03	−31.7	0.83	N/A

^a^Spectral library identification(m/z cloud)

^b^In silico identification(MetFrag)

^c^Precursor mass identification(hmdb/Metlin)

^d^hmdb(HMDB)/Metlin(MID) identifier code

**Table 2:** (a) Circulating metabolites increased in DIO mice treated daily with PEG-OB(Cys^10^, Cys^13^) for 6 weeks, (b) circulating metabolites decreased in DIO mice treated daily with PEG-OB(Cys^10^, Cys^13^) for 6 weeks

Putative ID	RT(min)_m/z	Formula	Adduct	Δ ppm	*P* value	FDR	% change	AUC (ROC)	Identifier^d^
(*a*)									
Amino acid and antioxidant related metabolites									
Creatine^a^	0.65_132.0763	C4H9N3O2	M + H	3	4.72E−06	1.18E−04	26.2	0.94	HMDB00064
Oxidised glutathione^b^	1.61_307.0828	C20H32N6O12S2	M + 2H	1	4.59E−02	4.78E−02	622.3	0.73	HMDB03337
Unknowns									
None	7.22_770.3435	None	N/A	N/A	1.67E−02	2.69E−02	761.8	0.86	N/A
None	6.17_599.6132	None	N/A	N/A	1.27E−02	2.29E−02	475.6	0.79	N/A
None	7.88_163.1329	None	N/A	N/A	9.83E−04	6.14E−03	39.1	0.74	N/A
None	7.08_820.8670	None	N/A	N/A	1.23E−02	2.29E−02	977.5	0.80	N/A
None	6.27_565.9308	None	N/A	N/A	1.22E−02	2.29E−02	427.2	0.75	N/A
(*b*)									
Phospholipids									
LysoPC(20:2(11Z,14Z))^b^	15.28_570.3528	C28H54NO7P	M + Na	0	2.96E−02	3.45E−02	−17.5	0.75	HMDB10392
LysoPC, LysoPE, PC or PE^c^	15.56_510.3558	C25H52NO7P	M + H	0	1.79E−02	2.69E−02	−6.1	0.76	Several
PAF C-18^b^	16.32_552.401	C28H58NO7P	M + H	2	1.10E−02	2.29E−02	−20.3	0.76	MID43410
PE(18:1(9Z)/0:0)^b^	15.42_480.3072	C23H46NO7P	M + H	2	3.64E−02	3.96E−02	−12.2	0.67	MID40778
LysoPE(0:0/20:2(11Z,14Z))^b^	15.30_528.3059	C25H48NO7P	M + Na	0	1.25E−03	6.27E−03	−32.1	0.82	HMDB11483
Fatty acid and fatty acyls									
7,10,13,16- Docosatetraenoic acid^b^	16.16_350.3062	C22H36O2	M + NH4	2	1.94E−02	2.69E−02	−13.6	0.74	HMDB02226
1-(*O*-α-d-glucopyranosyl)-29-keto-(1,3R,31R)-dotriacontanetriol^b^	17.11_675.5415	C38H74O9	M + H	1	1.28E−02	2.29E−02	−15.2	0.73	MID46604
*N*-oleoyl threonine^c^	15.98_348.2916	C22H41NO4	M + H-2H2O	2	7.76E−04	6.14E−03	−17.8	0.85	MID75490
Fatty acid, fatty acid ester, fatty alcohol or steroid ester^c^	16.15_333.28	C22H36O2	M + H	3	1.84E−02	2.69E−02	−18.8	0.75	Several
Monoacylglycerol, bile acid and unknowns									
MG(18:1(9Z)/0:0/0:0)^b^	15.80_339.2911	C21H40O4	M + H-H2O	3	2.55E−02	3.35E−02	−15.2	0.74	HMDB11567
Glycolithocholic acid^b^	14.81_478.2915	C26H43NO4	M + 2Na-H	2	9.69E−03	2.29E−02	−29.1	0.74	HMDB00698
None	0.91_169.0355	N/A	N/A	N/A	8.53E−03	2.29E−02	−12.4	0.78	N/A
None	11.64_244.1325	N/A	N/A	N/A	3.04E−02	3.45E−02	−98.6	0.69	N/A
None	10.02_530.8273	N/A	N/A	N/A	3.86E−05	4.82E−04	−33.9	0.89	N/A
None	15.80_357.3018	N/A	N/A	N/A	2.73E−02	3.42E−02	−14.6	0.73	N/A
None	16.07_374.3067	N/A	N/A	N/A	2.29E−03	8.18E−03	−28.6	0.82	N/A
None	7.58_449.2146	N/A	N/A	N/A	4.88E−02	4.88E−02	−33.3	0.70	N/A
None	15.98_331.2653	N/A	N/A	N/A	2.13E−03	8.18E−03	−23.9	0.81	N/A

**Fig. 4:**
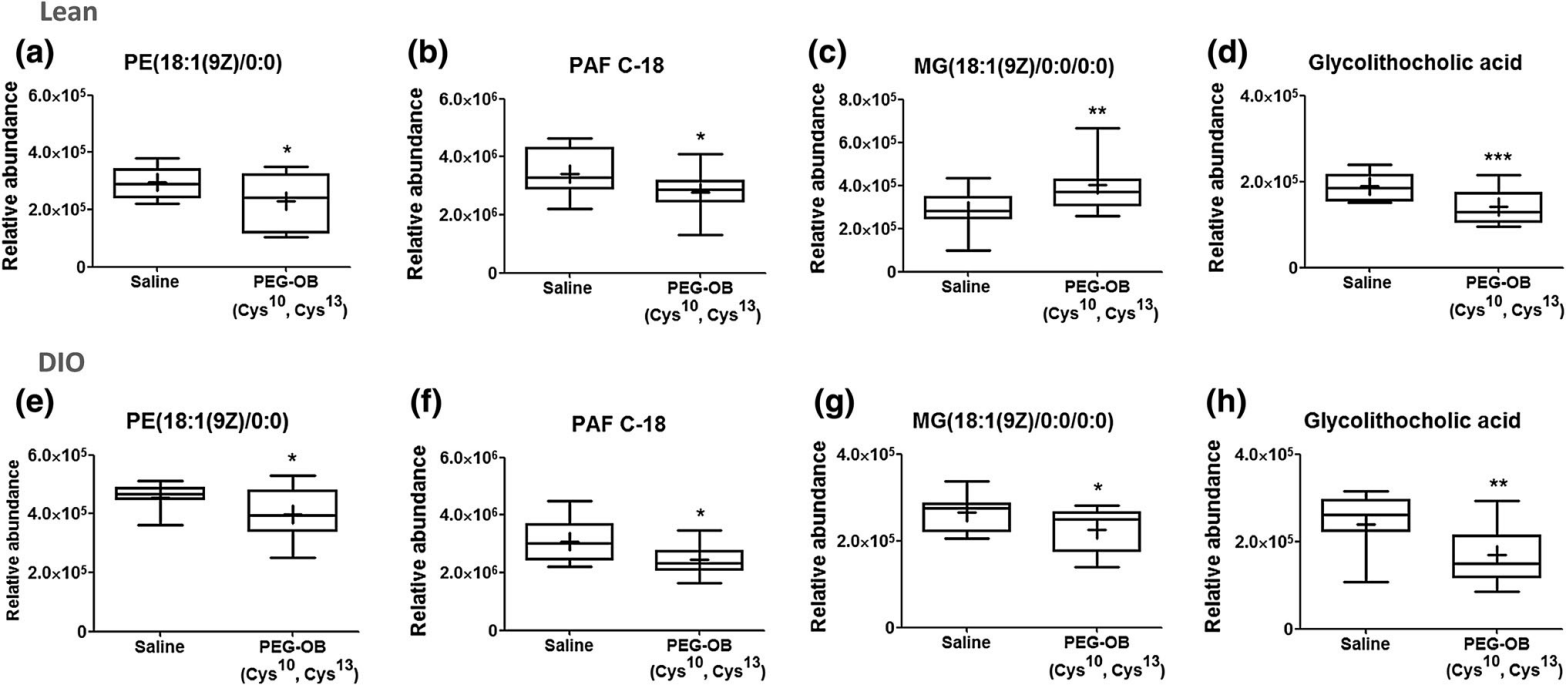
Box-and-whisker plots show the relative abundance of 4 circulating metabolites which were commonly affected by PEG-OB(Cys^10^,Cys^13^) treatment in lean mice (**a**–**d**) and DIO mice (**e**–**h**). Metabolites are **a**, **e** PE(18:1(9Z)/0:0), **b**, **f** PAF C-18, **c**, **g** MG(18:1(9Z)/0:0/0:0) and **d**, **h** glycolithocholic acid. *Whiskers* indicate min and max, *bottom* and *top* of *boxes* indicate 25th and 75th percentiles, and *line* through *centre* of each *box* and ‘+’ within *box* represent median and mean, respectively. *P* values were calculated by the Student’s *t* test with ***P < 0.001; **P < 0.01 and *P < 0.05 indicating significant differences between PEG-OB(Cys^10^,Cys^13^) treatment and saline

**Fig. 5:**
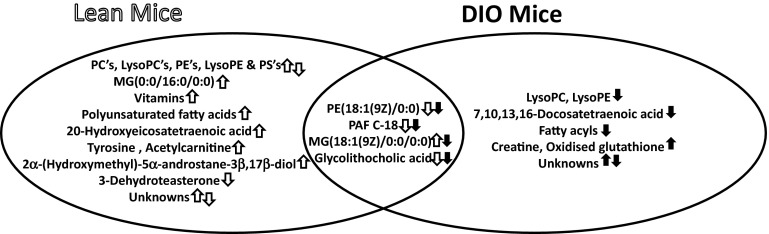
Venn diagram shows metabolites/metabolite classes increased and/or decreased in lean and DIO mice treated with PEG-OB(Cys^10^,Cys^13^). Directions of change are represented by *opened* and *closed arrows* for lean and DIO animals respectively

Across both groups, 3 metabolite identifications were assigned based on fragmentation pattern match with high resolution spectral library (m/z cloud) (available as online resources 4–6), and 40 metabolite identifications were putatively assigned based on in silico fragmentation match. Among those assigned, four were detected as altered between PEG-OB(Cys^10^, Cys^13^) and saline treated animals in both lean and DIO groups. Of these, two phospholipids (PE(18:1(9Z)/0:0) and PAF C-18), and a secondary bile acid (glycolithocholic acid) were decreased with treatment in both groups. A monoacylglycerol (MG(18:1(9Z)/0:0/0:0)) was also increased in lean mice and decreased in DIO mice (Fig. 4). When comparing PEG-OB(Cys^10^, Cys^13^) and saline treated lean mice, further metabolites were found to be present at increased levels and these comprised phospholipids (phosphatidylcholines (PC’s), a lysophosphatidylcholine (LysoPC), phosphatidylethanolamines(PE’s), and phosphatidylserines(PS’s)), monoacylglycerols, polyunsaturated fatty acids (α linolenic acid, arachidonic acid and metabolite) and vitamins (A, D and D derivatives). Moreover, tyrosine, acetylcarnitine and 2α-(hydroxymethyl)-5α-androstane-3β,17β-diol were also found to be elevated along with three unidentified features. Additional metabolites found to be at lower levels in lean mice were phospholipids (PC’s, PE’s, lysoPC’s and a lysoPE), 3-dehydroteasterone and one unidentified feature. PEG-OB(Cys^10^, Cys^13^) treated DIO animals showed increased levels of creatine, oxidised glutathione and five unidentified features when compared to saline treated controls. Conversely we identified more metabolites at lower levels in DIO treated animals. These consisted of phospholipids (PC’s, PE’s, lysoPC’s and lysoPE’s), as well as 1,7,10,13,16-docosatetraenoic acid, 1-(*O*-alpha-d-glucopyranosyl)-29-keto-(1,3R,31R)-dotriacontanetriol, *N*-oleoyl threonine, another metabolite of lipid class (a fatty acid, fatty acid ester, fatty alcohol or steroid ester by exact mass search in metlin/hmdb databases) and seven unidentified features (Fig. 5).

## Discussion

A variety of previous studies have provided evidence suggesting that obestatin has effects on metabolism. Whilst many have focused on effects on food intake and body weight, actions on glucose and lipid metabolism have also been investigated both under normal physiological conditions and in experimental models of obesity or diabetes (Agnew et al. 2011; Egido et al. 2009; Granata et al. 2008, 2010, 2012; Green et al. 2007; Nagaraj et al. 2014). In order to build on these data and to provide a detailed assessment of the metabolic actions of obestatin, the current study employed untargeted metabolomic analysis to assess whether obestatin influences the levels of circulating small molecule metabolites. In lean mice, treatment with the obestatin analogue was found to significantly affect 39 different metabolites, a finding which served as the basis for a follow-on investigation in DIO mice to understand if this peptide’s activity was dependent upon nutritional status. Importantly, the experimental protocol adopted for these lean and DIO studies was identical with the exception that DIO mice received a high fat (HF) diet for 13 weeks before commencing obestatin treatment at which time they displayed a mean bodyweight of 46 % higher than lean mice. To our knowledge, this is the first metabolomic investigation focussing on the actions of obestatin. Importantly, we employed a peptide analogue of obestatin (PEG-OB(Cys^10^, Cys^13^)) which demonstrates enhanced stability and preserved biological activity (Burch et al. 2015; Cowan et al. 2015). The results clearly show that obestatin treatment alters metabolite profiles in both lean and DIO mice. OPLS-DA analysis demonstrated clear separation of mice treated with PEG-OB(Cys^10^,Cys^13^) from saline controls (Fig. 2a, b) and this led to the shortlisting and identification of metabolite ions which were significantly altered (Tables 1, 2). It was immediately evident that lean and DIO mice responded differently to obestatin treatment. Of the 39 and 25 metabolites significantly modulated in lean and DIO mice, respectively, there were only 4 which were commonly affected in both groups. Of particular note, the majority of affected metabolites were increased in lean mice, whilst in contrast in DIO mice the majority of metabolite changes were decreased. Furthermore, regardless of whether metabolites were increased or decreased the overall magnitude of the responses was clearly greater in DIO mice (≤978 %) compared with lean mice (≤298 %). Interestingly, a large proportion of metabolites altered in DIO mice could not be putatively identified potentially suggesting the involvement of lesser known or uncharacterised biochemical pathways.

The four metabolites commonly affected in both lean and DIO mice included a phosphatidylethanolamine moiety (PE(18:1(9Z)/0:0)), a platelet activating factor (PAF C-18), a monoacylglycerol (MG(18:1(9Z)/0:0/0:0)) and a secondary bile acid (glycolithocholic acid). This clearly demonstrates a role for obestatin in lipid metabolism and suggests that some actions may be independent of nutritional status. These 4 metabolites were all significantly decreased in DIO mice (12–29 %) by PEG-OB(Cys^10^,Cys^13^) treatment, whilst in lean mice three were also decreased although one (MG(18:1(9Z)/0:0/0:0)) was elevated. Three of the above metabolites are biochemically similar to one another all being phospholipid molecules with a single 18 carbon chain. Interestingly, long chain phospholipids are negatively associated with obesity and diabetes and PAF is an inflammatory mediator which acts in part to promote cytokine production (Giesbertz et al. 2015; Li and Mitra 1996; Menezes-Garcia et al. 2014). Indeed, PAF receptor deficient mice fed a high carbohydrate diet have been shown to display reduced adipose tissue inflammation and improved glucose homeostasis (Menezes-Garcia et al. 2014), whilst other studies have reported increases in bile acid levels with HF feeding for relatively short periods (Sayin et al. 2013; Sun et al. 2015). Therefore, a key function of obestatin may be to control inflammation and to preserve healthy lipid and cholesterol metabolism both under normal physiological circumstances and also under situations of DIO/diabetes, and such actions could be mediated via the observed reductions in these particular metabolites. In support of this suggestion, obestatin has been shown to reduce obesity-induced increases in inflammatory cytokines, to decrease insulin sensitivity and to decrease triglyceride levels. Furthermore, it has also been shown to decrease the expression of cholesterol transporter ATP-binding cassette A1 (ABCA1) in bovine white adipose tissue (A. Agnew et al. 2011; Grala et al. 2010; Granata et al. 2012; Hotamisligil 2006).

Notably, the majority of circulating metabolites affected by PEG-OB(Cys^10^,Cys^13^) treatment in lean mice were phospholipids. Most of these were increased and most were species of PC, LysoPC, PE or PS, although there were also some instances of decreased PC, LysoPC, PE or LysoPE phospholipid species. This suggests that obestatin influences the turnover of phospholipids. Indeed, many plasma phospholipids originate from lipoproteins (Miller et al. 2011), and therefore obestatin’s ability to regulate phospholipid levels could be linked to its ability to regulate HDL, LDL and VLDL levels (Catak et al. 2014; Nagaraj et al. 2008, 2009). Other metabolites which were increased with PEG-OB(Cys^10^,Cys^13^) treatment in lean mice included monoacylglycerols, polyunsaturated fatty acids, vitamin A and D forms, an amino acid (tyrosine), an acylcarnitine (acetylcarnitine), a steroid (2α-(hydroxymethyl)-5α-androstane-3β,17β-diol) and some unknown metabolites. As acylcarnitines facilitate transport of fatty acids into the mitochondria for β-oxidation, an increase in acetylcarnitine could reflect decreased mitochondrial utilisation (Jüllig et al. 2007). Obestatin may therefore promote glucose utilisation over fatty acid utilisation for energy production when fatty acids are present under normal physiological conditions. This assertion is based on the observed increases in the circulating levels of polyunsaturated fatty acids and monoacylglycerols in PEG-OB(Cys^10^,Cys^13^) treated animals. It may even reflect a tendency for obestatin to safeguard against potential hyperglycaemia. Interestingly, physiological levels of polyunsaturated fatty acids have been associated with decreased and increased proinflammatory and anti-inflammatory markers, respectively (Ferrucci et al. 2006). Therefore, slightly elevated levels observed in lean mice treated with PEG-OB(Cys^10^, Cys^13^) further corroborate a role for obestatin in limiting inflammation. In addition, the observed increases in 2α-(hydroxymethyl)-5α-androstane-3β,17β-diol, vitamin D3 and vitamin A metabolites could also be viewed as beneficial. 2α-(hydroxymethyl)-5α-androstane-3β,17β-diol is a metabolite of dehydroepiandrosterone (DHEA) and dihydrotestosterone (DHT) and both insulin resistance and obesity have been associated with low androgen and vitamin D levels (Navarro et al. 2015; Pereira-Santos et al. 2015). Furthermore, vitamin A is known to improve insulin sensitivity and has been suggested essential for maintenance of pancreatic β-cell function and mass (Jeyakumar et al. 2011; Trasino et al. 2015).

Numerous studies have associated increased levels of the amino acid tyrosine with diabetes and obesity, although a few have reported decreased levels in this setting (Mihalik et al. 2012; Tranchida et al. 2015; X. Zhang et al. 2009). Furthermore, 3-dehydroteasterone is a sterol lipid of dietary source and a decreased level of this metabolite with PEG-OB(Cys^10^,Cys^13^) treatment in animals maintained on the same diet suggests that obestatin may inhibit its uptake from the gut.

Of the seven metabolites increased with PEG-OB(Cys^10^,Cys^13^) treatment in DIO mice only two could be putatively identified: creatine and oxidised glutathione (GSSG). GSSG is the oxidised form of the antioxidant reduced glutathione (GSH), and increased GSSG and GSSG/GSH ratio are considered to be indicative of oxidative stress and are associated with obesity and diabetes (Savini et al. 2013). However, it is not clear what effect PEG-OB(Cys^10^, Cys^13^) treatment has on the glutathione system, when the observed increase in GSSG may also be explained by sample storage conditions. Indeed, GSH is known to degrade at a much faster rate than GSSG, and is also susceptible to autoxidation (Lin et al. 2006); therefore, GSSG measured here may not reflect true plasma GSSG levels.

Increased plasma creatine could suggest decreased creatine uptake by skeletal muscle, and interestingly, skeletal muscle absorption of creatine is believed to be promoted by insulin (Steenge et al. 1998). The increased creatine levels observed here may thus reflect decreased insulin production with PEG-OB(Cys^10^, Cys^13^) treatment. Furthermore, hyperinsulinemia is one of the hallmarks of insulin resistance and thus obestatin could play a protective role against the development of insulin resistance. Indeed, supportive evidence for this suggestion is provided by a report that creatine levels decrease whilst glucose and insulin levels increase in HF fed mice (Duggan et al. 2011).

In addition to those already discussed, further metabolites found to decrease with PEG-OB(Cys^10^, Cys^13^) treatment in DIO mice included polyunsaturated fatty acids and fatty acyls. Significantly increased levels of fatty acids are well documented in obesity and diabetes, and so reduced polyunsaturated fatty acids and fatty acyls with PEG-OB(Cys^10^, Cys^13^) treatment in DIO animals suggests that obestatin has the potential to protect against lipid dysregulation under these conditions (Lopaschuk et al. 2010). Such protective effects of obestatin could involve decreasing adipose tissue lipolysis (Granata et al. 2012) and/or increasing fatty acid clearance by β-oxidation.

Altered levels of plasma lipoproteins (the source of phospholipids) in plasma are linked to inflammation, β-cell dysfunction, obesity and diabetes (Anderson et al. 2014; Miller et al. 2011; Roehrich et al. 2003). LysoPC’s are major components of oxidized low density lipoproteins (Ox-LDL), and not surprisingly increased levels have been correlated with endothelial dysfunction and atherogenesis, as well as inflammation and oxidative stress (Aiyar et al. 2007; Galili et al. 2007; Schmitz and Ruebsaamen 2010). Furthermore, phospholipids have been reported to be susceptible to glycoxidation and are suggested to be a potential source of oxidative damage to tissues (Miyazawa et al. 2008; Podrez 2002). The ability of PEG-OB(Cys^10^, Cys^13^) treatment to reduce high phospholipid levels in plasma of DIO animals therefore suggests that obestatin may be able to ameliorate lipoprotein/phospholipid mediated proinflammatory stress associated with complications of obesity and/or diabetes.

Interestingly, as previously discussed, longer rather than shorter chain phospholipids have been negatively associated with obesity and diabetes, and the hypoglycaemic effect of insulin has been shown to improve when phospholipids of reducing carbon numbers from C18 to C10 are involved (Giesbertz et al. 2015; Li and Mitra 1996). Therefore, reduction of phospholipids with carbon lengths of ≥18 as observed here in DIO mice treated with PEG-OB(Cys^10^, Cys^13^) suggests that obestatin may be promoting insulin absorption/sensitivity by reducing the ratio of longer to shorter carbon chain phospholipids.

## Concluding remarks

In summary, this UPLC-MS metabolomic investigation has elucidated a number of plasma metabolite changes which occur as a consequence of obestatin treatment. In lean mice 39 metabolites were significantly affected, strongly suggesting that obestatin modulates metabolism and thereby appearing to justify its proposed status as a hormone.

The fact that there were 4 metabolites commonly affected in DIO and lean mice demonstrates that there is at least some consistency in their responses to obestatin. However, there were a further 21 unique metabolite responses in DIO mice, clearly showing that nutritional status has a bearing on obestatin’s mode of action. The metabolite changes observed here suggest that this peptide influences lipid metabolism and may also play an anti-inflammatory role. Furthermore, some of the observed metabolite changes may underlie the previously reported beneficial actions of obestatin on insulin resistance, hyperglycaemia and lipid dysregulation. Together, the findings of this study clearly highlight the potential value of metabolomics to delineate the metabolic actions of poorly characterised physiological hormones.

## Electronic supplementary material

Below is the link to the electronic supplementary material.

Supplementary Fig. 1 PCA scores plot displaying pools (red triangles), samples from lean and DIO mice (black triangles) and samples from another study (grey triangles; data not presented)Supplementary material 1 (PDF 237 kb)

Supplementary Fig. 2 PCA scores plot displaying the separation for lean mice (a) and DIO mice (b) treated daily with PEG-OB(Cys^10^,Cys^13^) (triangles) or with saline (circles) for 6 weeks. Explained variances (R2) were 0.978 and 0.854 and predictive abilities (Q2) were 0.861 and 0.742 for lean and DIO mice, respectively (PDF 164 kb)

Supplementary Fig. 3 OPLS-DA scores plot displaying the separation for lean mice (a) and DIO mice (b) treated daily with PEG-OB(Cys^10^, Cys^13^) (triangles) or with saline (circles) for 6 weeks. Explained variances (R2) were 0.983 and 0.969 and predictive abilities (Q2) were 0.948 and 0.932 for lean and DIO mice respectively. Corresponding validation plots for lean mice (c) and DIO mice (d) displaying 999 permutation tests for model built from PEG-OB(Cys^10^,Cys^13^) treated animals (PDF 249 kb)

Supplementary Fig. 4 Detection and identification of creatine. MS^2^ mass spectrum obtained at 0.15–1.15 min (a), corresponding MS^2^ peak at 0.15–1.15 min (b) and high resolution spectral library match in m/z cloud (reference no 357)(c) (PDF 189 kb)

Supplementary Fig. 5 Detection and identification of acetylcarnitine. MS^2^ mass spectrum obtained at 0.45–1.44 min (a), corresponding MS^2^ peak at 0.45–1.44 min (b), and high resolution spectral library match in m/z cloud (reference no 879)(c) (PDF 187 kb)

Supplementary Fig. 6 Detection and identification of 9-cis retinal. MS^2^ mass spectrum obtained at 13.83–14.82 min (a), corresponding MS^2^ peak at 13.83–14.82 min (b), and high resolution spectral library match in m/z cloud (reference no 836)(c) (PDF 191 kb)
