# Supplementary material for: Treatment of lean and diet-induced obesity (DIO) mice with a novel stable obestatin analogue alters plasma metabolite levels as detected by untargeted LC–MS metabolomics
Source: Metabolomics. 2016 Jul 5;12:124. doi: 10.1007/s11306-016-1063-0 (PMC4932145; doi:10.1007/s11306-016-1063-0)

Supplementary figure 1

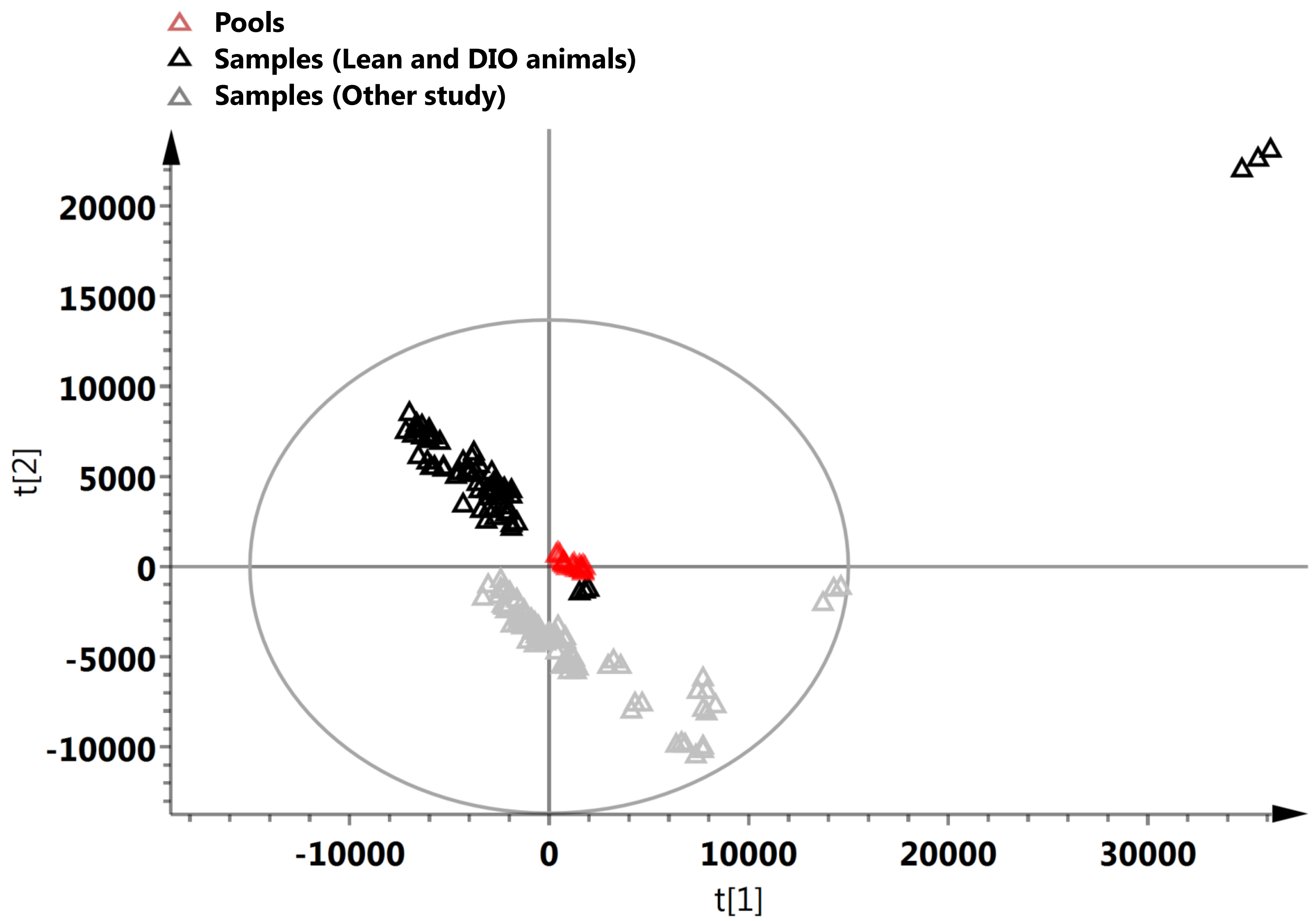

Supplement: Supplementary file 1 — PCA scores plot displaying pools (red triangles), samples from lean and DIO mice (black triangles) and samples from another study (grey triangles; data not presented)Supplementary material 1 (PDF 237 kb) [file 11306_2016_1063_MOESM1_ESM.pdf]
