# Supplementary figures and images for: Treatment of lean and diet-induced obesity (DIO) mice with a novel stable obestatin analogue alters plasma metabolite levels as detected by untargeted LC–MS metabolomics
Source: Metabolomics. 2016 Jul 5;12:124. doi: 10.1007/s11306-016-1063-0 (PMC4932145; doi:10.1007/s11306-016-1063-0)

## Supplementary figure 2

(a) Lean

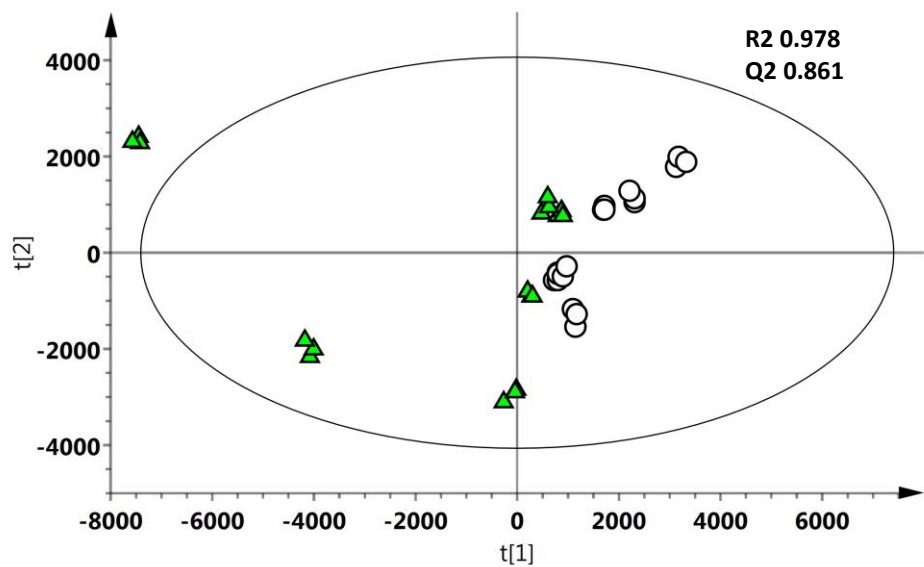

(b) DIO

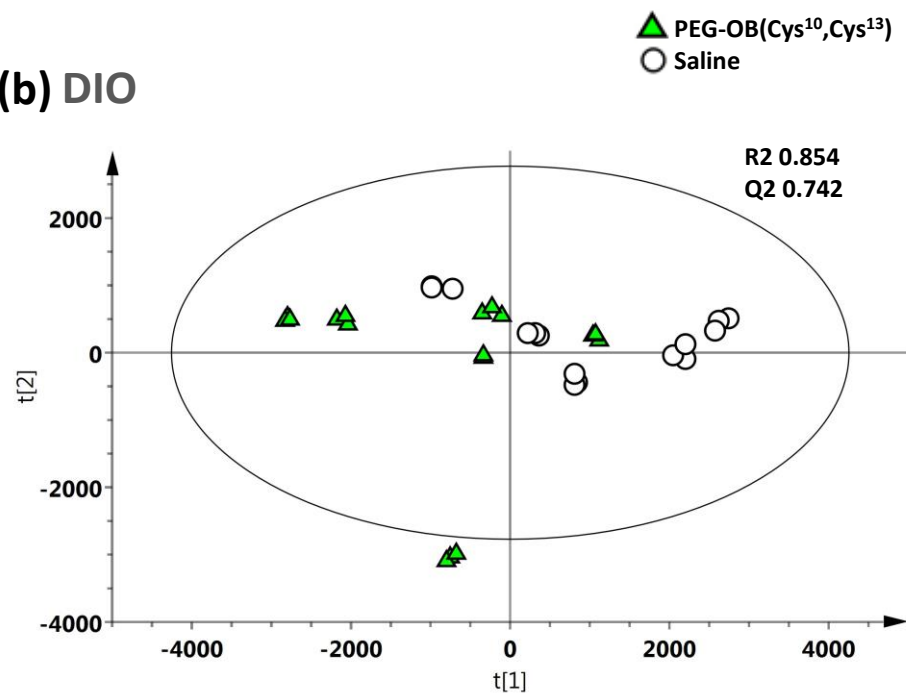

Supplement: Supplementary file 2 — PCA scores plot displaying the separation for lean mice (a) and DIO mice (b) treated daily with PEG-OB(Cys10,Cys13) (triangles) or with saline (circles) for 6 weeks. Explained variances (R2) were 0.978 and 0.854 and predictive abilities (Q2) were 0.861 and 0.742 for lean and DIO mice, respectively (PDF 164 kb) [file 11306_2016_1063_MOESM2_ESM.pdf]

Supplementary figure 3

(a) Lean

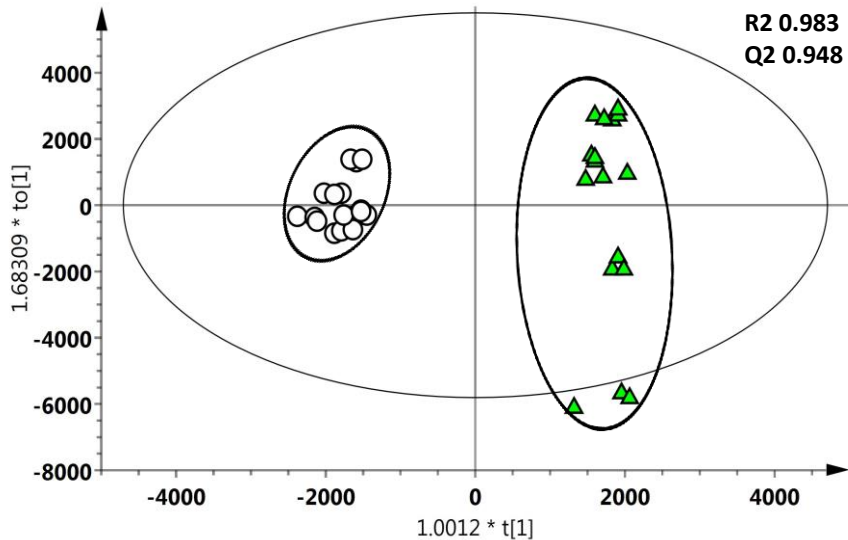

(b) DIO

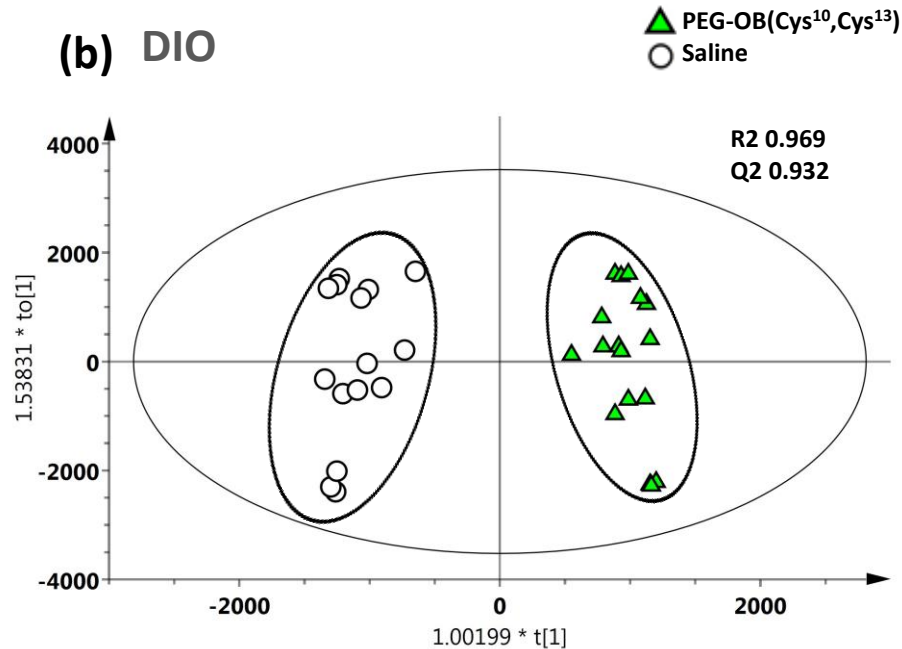

(c) Lean

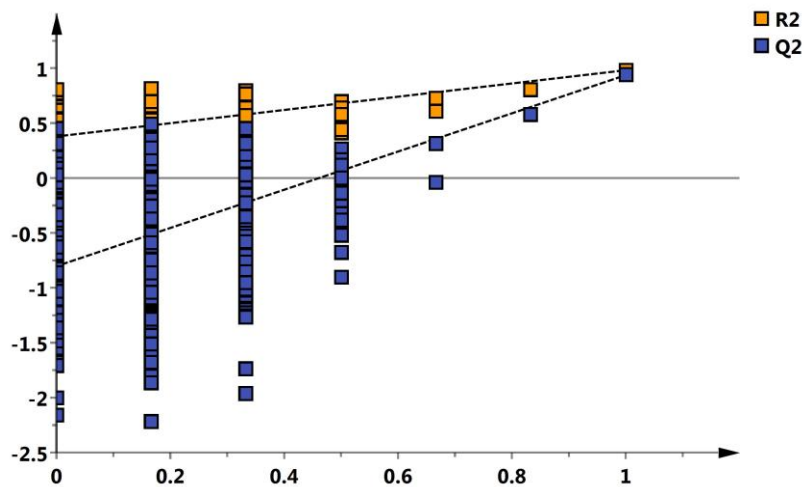

(d) DIO

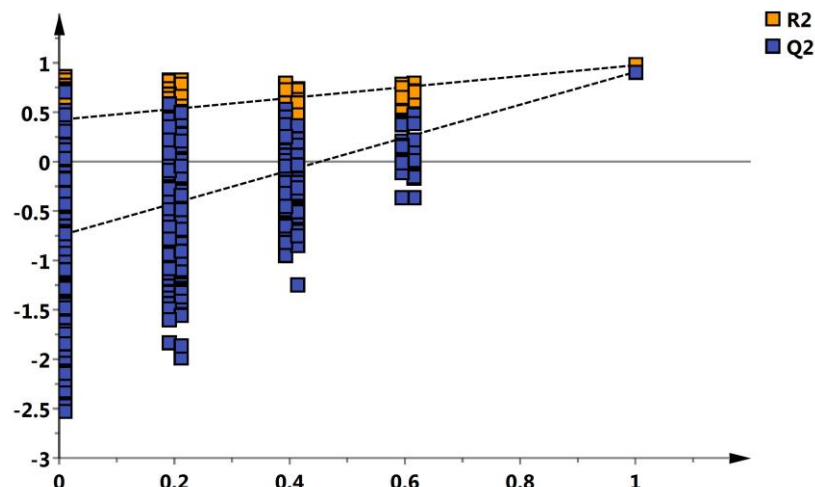

Supplement: Supplementary file 3 — OPLS-DA scores plot displaying the separation for lean mice (a) and DIO mice (b) treated daily with PEG-OB(Cys10, Cys13) (triangles) or with saline (circles) for 6 weeks. Explained variances (R2) were 0.983 and 0.969 and predictive abilities (Q2) were 0.948 and 0.932 for lean and DIO mice respectively. Corresponding validation plots for lean mice (c) and DIO mice (d) displaying 999 permutation tests for model built from PEG-OB(Cys10,Cys13) treated animals (PDF 249 kb) [file 11306_2016_1063_MOESM3_ESM.pdf]

## Supplementary figure 4

(a)

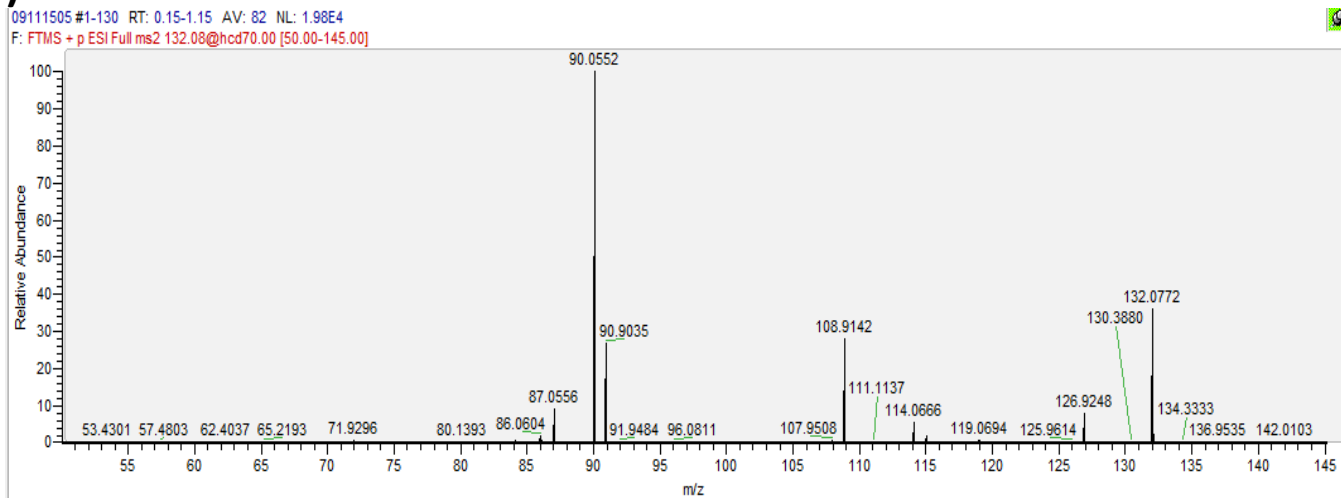

(b)

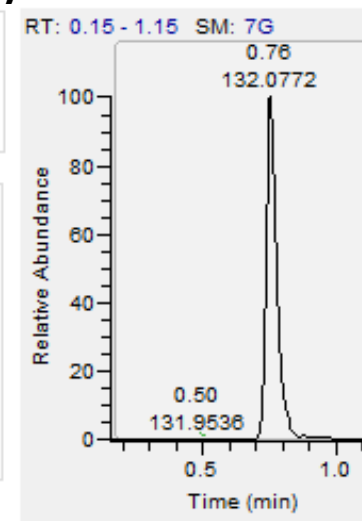

(c)

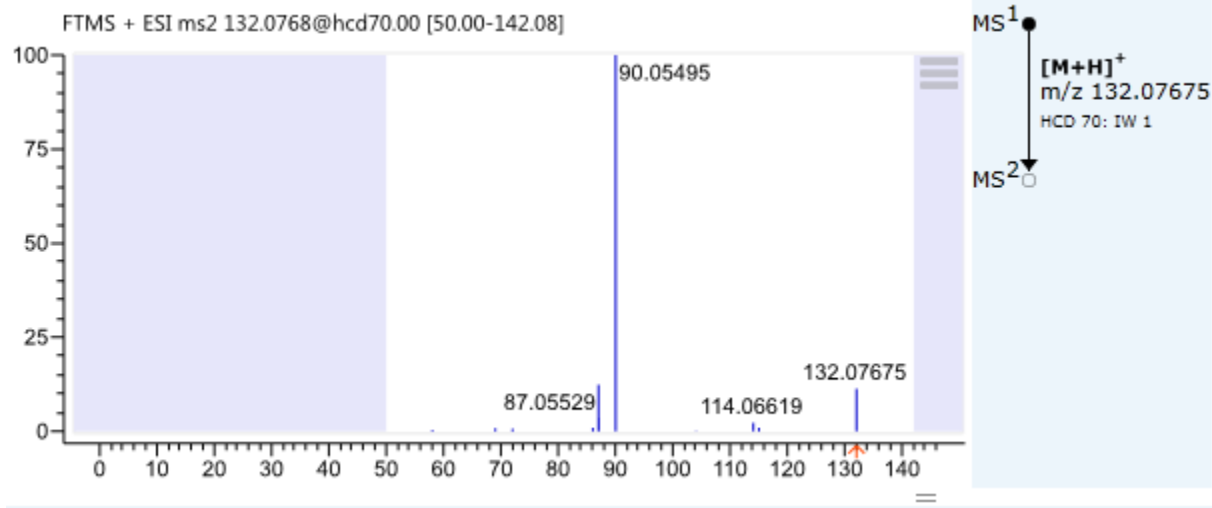

Supplement: Supplementary file 4 — Detection and identification of creatine. MS2 mass spectrum obtained at 0.15–1.15 min (a), corresponding MS2 peak at 0.15–1.15 min (b) and high resolution spectral library match in m/z cloud (reference no 357)(c) (PDF 189 kb) [file 11306_2016_1063_MOESM4_ESM.pdf]

## Supplementary figure 5

(a)

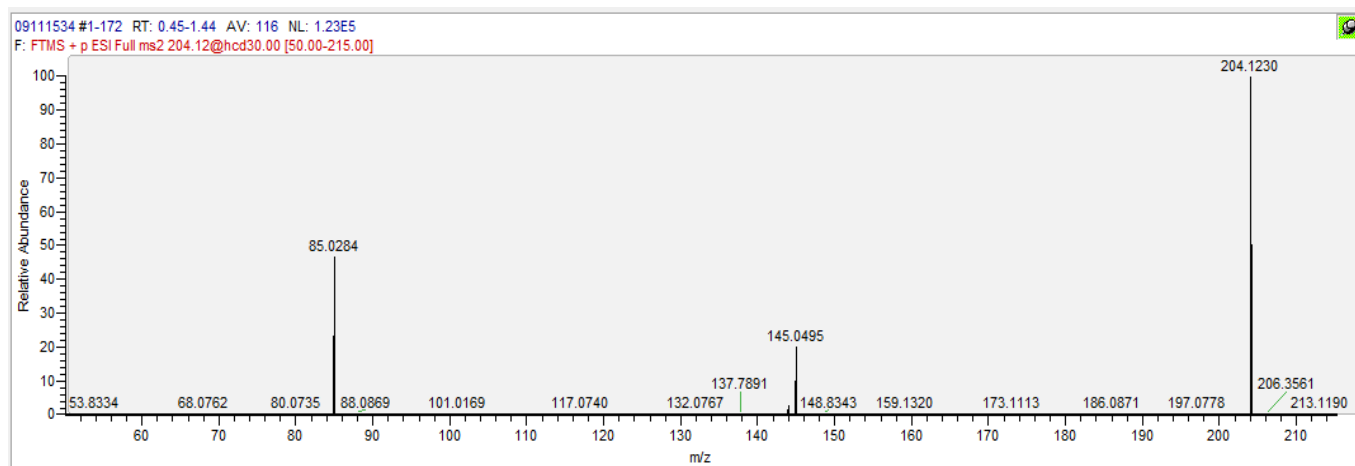

(b)

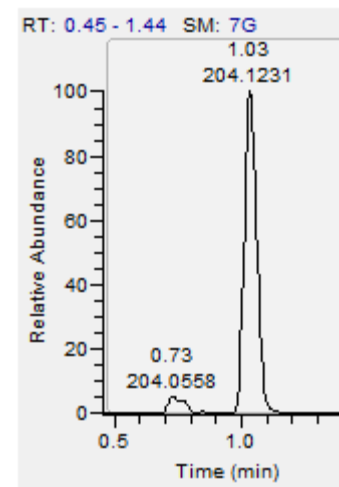

(c)

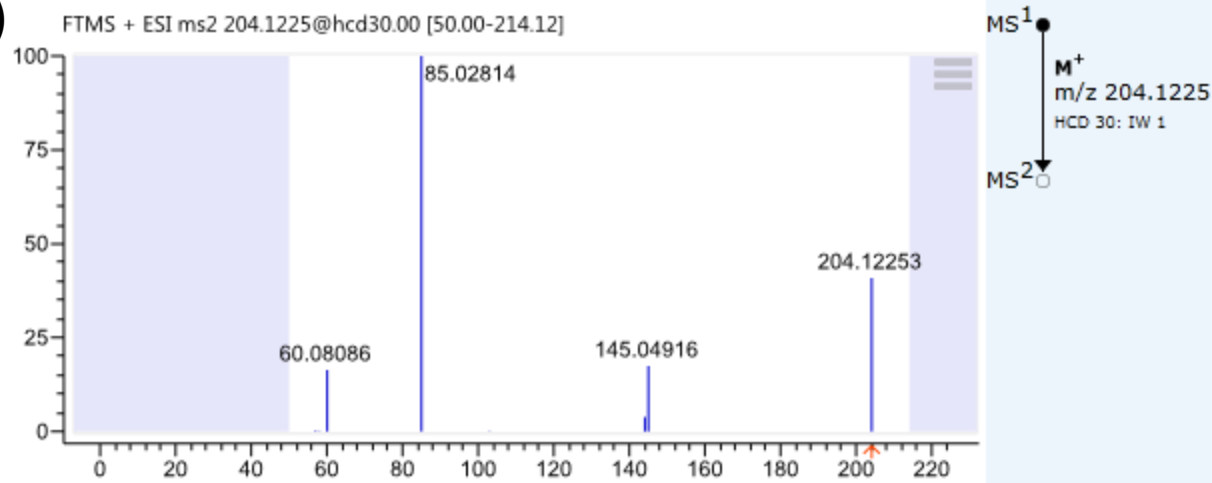

Supplement: Supplementary file 5 — Detection and identification of acetylcarnitine. MS2 mass spectrum obtained at 0.45–1.44 min (a), corresponding MS2 peak at 0.45–1.44 min (b), and high resolution spectral library match in m/z cloud (reference no 879)(c) (PDF 187 kb) [file 11306_2016_1063_MOESM5_ESM.pdf]

## Supplementary figure 6

(a)

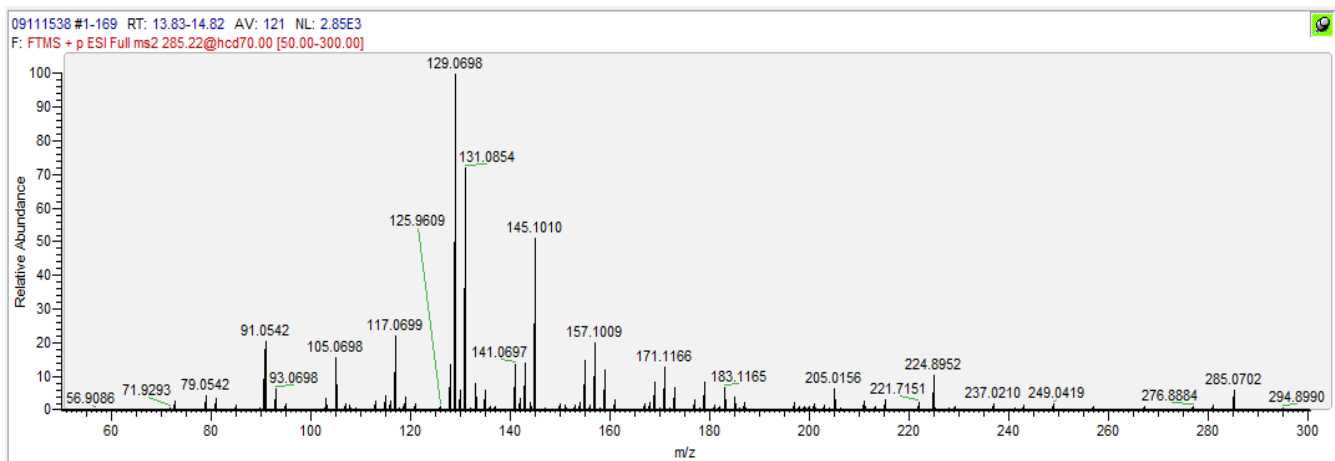

(b)

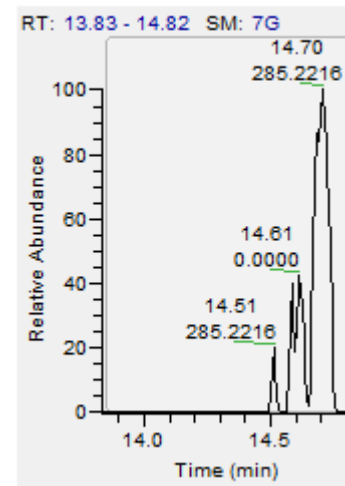

(c)

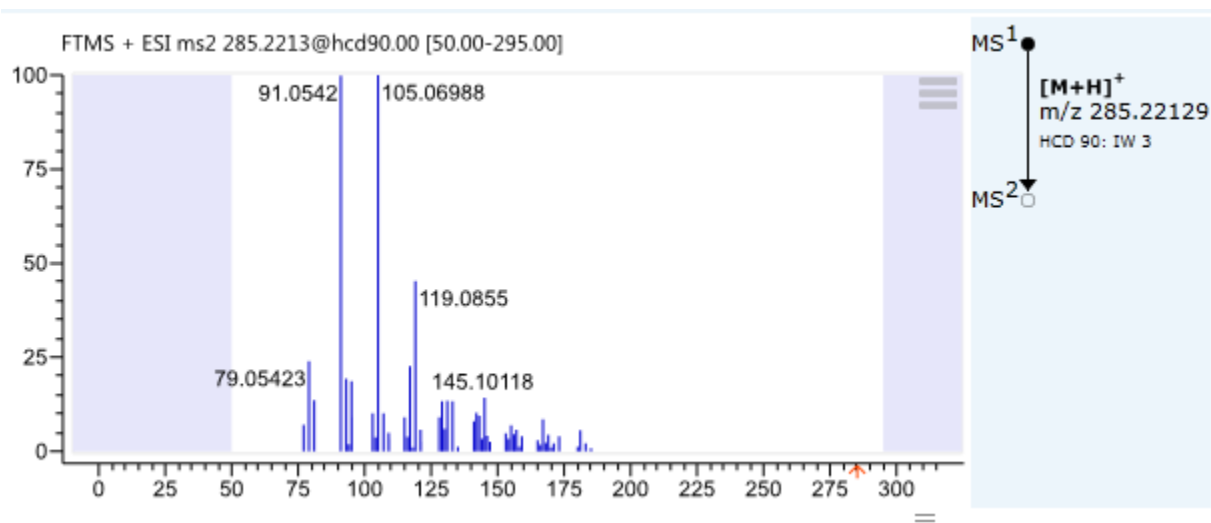

Supplement: Supplementary file 6 — Detection and identification of 9-cis retinal. MS2 mass spectrum obtained at 13.83–14.82 min (a), corresponding MS2 peak at 13.83–14.82 min (b), and high resolution spectral library match in m/z cloud (reference no 836)(c) (PDF 191 kb) [file 11306_2016_1063_MOESM6_ESM.pdf]
